# Supplementary material for: Mpox Incidence and Vaccine Uptake in Men Who Have Sex with Men and Are Living with HIV in Denmark
Source: Vaccines (Basel). 2023 Jun 27;11(7):1167. doi: 10.3390/vaccines11071167 (PMC10385255; doi:10.3390/vaccines11071167)
Supplement: Supplementary file 1 [file vaccines-11-01167-s001.zip › vaccines-2462689-supplementary.pdf]

Supplementary Materials

Table S1. Missing data for clinical characteristics and survey responses

**All participants**<br>**n = 227**	**Missing data**<br>**n (%)**
**Age (years)**	0 (0%)
**Men**	0 (0%)
**Alcohol (grams per week)**	0 (0%)
**Alcohol more than 10 units per week**	0 (0%)
**Smoking status**	11 (1.5%)
**Origin**	0 (0%)
**Plasma HIV RNA < 50 copies/mL**	0 (0%)
**Blood $CD4^+$ T lymphocyte count, cells/μL**	127 (17.5%)
**Education**	31 (4.3%)
**STD in the preceding two years**	0 (0%)

Participants who answered the online survey<br><br>n = 401	Missing data<br><br>n (%)
**Have you heard about mpox?**	2 (0.5%)
**Do you see yourself as being at risk of the mpox infection?**	2 (0.5%)
**Have you heard about the mpox vaccine?**	11 (2.7%)
**Have you been offered the vaccine?**	12 (3%)
**Have you received the mpox vaccine?**	11 (2.7%)
**Why did you choose to get the vaccine?**	0 missing
**Why did you choose not to get the vaccine?**	0 missing
**On a scale of 1-5 how willing are you to get the mpox vaccine?**	38 (9.5%)
**On a scale of 1-5 how worried are you about getting infected with mpox?**	6 (1.5%)

**How many sexual partners have you had in the last 12 weeks?**	11 (2.7%)
**Have you received the smallpox vaccine as a child?**	12 (3%)

STD, sexually transmitted disease

Table S2. Prediction of incident mpox using Cox regression

**Unadjusted**	Hazard Ratio	P-value
Age (per year)	0.9, 95% CI 0.8-0.9	0.01
STD in the preceding 2 years	10.1, 95% CI 2.8-36.6	<0.001
Alcohol (units per week)	0.9, 95% CI 0.9-1.0	0.2
Alcohol more than 10 units per week	0.6, 95% CI 0.2-2.0	0.4
Undetectable viral load	0.7, 95% CI 0.1-5.6	0.8
**Model 1**	Hazard Ratio	P-value
Age (per year)	0.95, 95% CI 0.9-1.0	0.1
STD in the preceding 2 years	7.1, 95% CI 1.9-26.9	0.004

Model 1 is adjusted for age and a diagnosis with at least one STD in the preceding two years. STD, sexually transmitted disease

Table S3. Univariable and multivariable logistic regression of the factors associated with vaccine uptake among MSM LWH

	**Mpox vaccination uptake**					
	**Univariable model**			**Multivariable model**		
	**OR**	**95%CI**	**p-value**	**aOR**	**95%CI**	**p-value**
**Age (per year)**	0.9	0.9-1.0	0.003	0.9	0.9-1.0	0.2
**STD in the preceding 2 years**	3.4	2.4-7.8	<0.001	3.1	2.2-4.6	<0.001
**Alcohol (units per week)**	1.0	1.0-1.0	0.2			
**Alcohol more than 10 units per week**	0.9	0.7-1.3	0.6			
**Smoking status**						
Never smoker	Ref.			Ref.		

Ex-smoker	0.8	0.6-1.2	0.21	0.9	0.6-1.4	0.8
Current smoker	0.6	0.4-0.9	0.01	0.6	0.4-0.9	0.01
**Origin**						
Scandinavian	Ref.					
Other	0.9	0.5-1.4	0.6			
Other European	0.7	0.4-1.2	0.2			
**Education**						
Long education	Ref.					
No education or short education	0.8	0.6-1.2	0.3			
**Undetectable viral load**	0.8	0.4-1.4	0.4			
**CD4**	1.0	1.0-1.0	0.6			

OR, odds ratio; CI, confidence interval; aOR, adjusted odds ratio; STD, sexually transmitted disease Multivariable model is adjusted for age, STD and smoking status.

#	Variable / Field Name	Field Label<br>*Field Note*	Field Attributes (Field Type, Validation, Choices, Calculations, etc.)
Instrument: **Spørgeskema om Vaccination mod Abekopper** (sprgeskema_om_vaccination_mod_abekopper) Enabled as survey			
1	[participant_id]	Participant ID	text
2	[consent]	Tak fordi, du har valgt at deltage i spørgeskemaundersøgelsen. I det følgende spørgeskema vil vi still dig nogle spørgsmål om abekopper og vaccination. Ved at klikke ja giver du samtykke til, vi må bruge dine oplysninger fra COCOMO-studiet.	radio<br>1 Ja
3	[cpr_0]<br>Show the field ONLY if:<br>[consent] = '1'	Ved at udfylde dit CPR-nummer har vi mulighed for at sammenkoble din besvarelse med blodprøvesvar.	descriptive
4	[cpr]<br>Show the field ONLY if:<br>[consent] = '1'	Indtast dit CPR-nummer	text (CPR_danish)
5	[nocpr]<br>Show the field ONLY if:<br>[consent] = '1'	Jeg ønsker ikke at udfylde CPR-nummer	radio<br>0 Jeg ønsker ikke at udfylde CPR-nummer
6	[q1]<br>Show the field ONLY if:<br>[consent] = '1'	Section Header: *Spørgsmål om abekopper også kaldet monkeypox*<br>Har du hørt om abekopper?	radio, Required<br>1 Ja<br>0 Nej<br>9 Ved ikke / ønsker ikke at svare<br>Question number: 1
7	[q2]<br>Show the field ONLY if:<br>[consent] = '1'	Ser du dig selv som værende i risikogruppen for at få abekopper?	radio, Required<br>1 Ja<br>0 Nej<br>9 Ved ikke / ønsker ikke at svare<br>Question number: 2
8	[monkeypox]	På en skala fra 1-5, hvor 1 er 'slet ikke bekymret' og 5 er 'ekstremt bekymret'. Hvor bekymret er du for at få abekopper?	radio (Matrix)<br>1 1<br>2 2<br>3 3<br>4 4<br>5 5<br>9 Ved ikke/Ønsker ikke at svare<br>Question number: 3
9	[q3]<br>Show the field ONLY if:<br>[consent] = '1'	Section Header: *Spørgsmål om vaccine mod abekopper*<br>Har du hørt om vaccinen mod abekopper?	radio, Required<br>1 Ja<br>0 Nej<br>9 Ved ikke / ønsker ikke at svare<br>Question number: 4
10	[vaccine]<br>Show the field ONLY if:<br>[q3] = '1' or [q3] = '9'	På en skala fra 1-5, hvor 1 er 'slet ikke villig' og 5 er 'ekstremt villig'. Hvor villig er du til at få vaccinen mod abekopper?	radio (Matrix)<br>1 1<br>2 2<br>3 3<br>4 4<br>5 5<br>9 Ved ikke/Ønsker ikke at svare<br>Question number: 5
11	[q_partners]	Hvor mange seksualpartnere har du haft i de sidste 12 uger?	text (number, Min: 0), Required<br>Question number: 6

12	[q4]<br>Show the field ONLY if:<br>[consent] = '1'	Er du blevet tilbudt vaccinen mod abekopper?	radio, Required<br>1 Ja<br>0 Nej<br>9 Ved ikke / ønsker ikke at svare<br>Question number: 7
13	[q5]<br>Show the field ONLY if:<br>[consent] = '1'	Har du fået vaccinen mod abekopper?	radio, Required<br>1 Ja<br>0 Nej<br>9 Ved ikke / ønsker ikke at svare<br>Question number: 8
14	[q6]<br>Show the field ONLY if:<br>[q5] = '1'	Hvorfor valgte du at takke ja til vaccinen mod abekopper?	checkbox, Required<br>1 q6___1 Jeg ser mig selv som værende i risikogruppen<br>2 q6___2 Jeg er bekymret for konsekvenserne af at blive smittet med abekopper<br>3 q6___3 Jeg har fået det anbefalet af en sundhedsfaglig<br>4 q6___4 Jeg har fået den anbefalet af en ven/familiemedlem/partner<br>5 q6___5 Andet<br>9 q6___9 Ved ikke / ønsker ikke at svare<br>Question number: 9
15	[q6comment]<br>Show the field ONLY if:<br>[q6(5)] = '1'	Du har svaret "Andet". Beskriv venligst nærmere	notes
16	[q7]<br>Show the field ONLY if:<br>[q5] = '0'	Hvorfor valgte du at takke nej til vaccinen mod abekopper?	checkbox, Required<br>1 q7___1 Jeg har allerede haft abekopper<br>2 q7___2 Jeg ser ikke mig selv som værende i risikogruppen<br>3 q7___3 Jeg tror ikke på, at vaccinen er effektiv<br>4 q7___4 Jeg gør brug af alle de nødvendige forebyggende metoder for ikke at blive smittet med abekopper<br>5 q7___5 Jeg har ikke hørt om vaccinen<br>7 q7___7 Jeg er allergisk<br>6 q7___6 Andet<br>9 q7___9 Ved ikke / ønsker ikke at svare<br>Question number: 9
17	[q7comment]<br>Show the field ONLY if:<br>[q7(6)] = '1'	Du har svaret "Andet". Beskriv venligst nærmere	notes
18	[variola]<br>Show the field ONLY if:<br>[consent] = '1'	Har du som barn fået koppevaccinen?"	radio, Required<br>1 Ja<br>0 Nej<br>9 Ved ikke / ønsker ikke at svare<br>Question number: 10
19	[sprgeskema_om_vaccination_mod_abekopper_complete]	Section Header: *Form Status*<br>Complete?	dropdown<br>0 Incomplete<br>1 Unverified<br>2 Complete

Figure S1. Mpox vaccine survey

Mpox vaccine survey translated to English.

Question 1: Have you heard about mpox? (yes/no)

Question 2: Do you see yourself as being at risk of the mpox infection? (yes/no)

Question 3: Have you heard about the mpox vaccine? (yes/no)

Question 4: Have you been offered the vaccine against mpox? (yes/no)

Question 5: Have you received the mpox vaccine? (yes/no)

Question 6: Why did you choose to get the vaccine?

- I see myself as being in the risk group
- I am worried about the consequences of being infected with mpox
- It was recommended by a health care professional
- It was recommended by a friend/family member/partner
- Other reason

Question 7: Why did you choose not to get the vaccine?

- I have already had mpox
- I am not seeing myself as being in the risk group
- I don't believe in the efficiency of the vaccine
- I'm using all other sorts of protecting measures in order not to get infected with mpox
- I have not heard about the vaccine
- I am allergic
- Other reason
- Don't know/don't wish to answer

Question 8: On a scale of 1-5, where 1 is "not worried at all" and 5 is "very worried", how worried are you about getting infected with mpox? (1-5)

Question 9: On a scale of 1-5, where 1 is "not willing at all" and 5 is "very willing", how willing are you to get the mpox vaccine? (1-5)

Question 10: How many sexual partners have you had in the last 12 weeks?

Question 11: Have you received the smallpox vaccine as a child?
